# Facile synthesis of NiCo_2_S_4_/CNTs nanocomposites for high-performance supercapacitors

**DOI:** 10.1098/rsos.180953

**Published:** 2018-09-12

**Authors:** Yunxia Huang, Ming Cheng, Zhongcheng Xiang, Yimin cui

**Affiliations:** Department of Physics, Beihang University, Beijing 100191, People's Republic of China

**Keywords:** porous NiCo_2_S_4_/CNTs, supercapacitor, different sulfide sources, cycling stability

## Abstract

Herein, porous NiCo_2_S_4_/CNTs nanocomposites were synthesized via a simple hydrothermal method followed by the sulphurization process using different sulfide sources. By comparing two different sulfur sources, the samples using thioacetamide as sulfide source delivered more remarkable electrochemical performance with a high specific capacitance of 1765 F g^−1^ at 1 A g^−1^ and an admirable cycling stability with capacitance retention of 71.7% at a high current density of 10 A g^−1^ after 5000 cycles in 2 M KOH aqueous electrolyte. Furthermore, an asymmetric supercapacitor (ASC) device was successfully fabricated with the NiCo_2_S_4_/CNTs electrode as the positive electrode and graphene as the negative electrode. The device provided a maximum energy density of 29.44 W h kg^−1^ at a power density of 812 W kg^−1^. Even at a high power density of 8006 W kg^−1^, the energy density still reaches 16.68 W h kg^−1^. Moreover, the ASC presents 89.8% specific capacitance retention after 5000 cycles at 5 A g^−1^. These results reveal its great potential for supercapacitors in electrochemical energy storage field.

## 1. Introduction

In recent years, it is urgently needed to develop clean and renewable energy resources due to environmental pollution and depletion of fossil fuels [1–5]. Therefore, a large number of energy conversion/storage devices have been explored to meet the increasing demand for energy and power in our daily life [6–8]. Among them, supercapacitors have received considerable attention on account of their high power density, low cost, fast charge–discharge rate, and long cycling life [9–12]. Particularly, pseudocapacitors, which make use of reversible Faradaic reactions occurring at the electrode surface to offer much higher specific capacitance, have become a research hotspot [10,13].

Transition metal oxides, hydroxides and their compounds are becoming widely explored for high-performance pseudocapacitors because of their low cost, low toxicity and great flexibility in structure and morphology [14,15]. However, their conductivity is typically too low to support fast electron transport towards high rate capability [16,17]. To overcome this problem, many substitute electrode materials have been developed. Among these substitute materials, transition metal sulfides have received considerable attention, due to their enhanced electrical conductivity and electrochemical activity compared with their corresponding transition metal oxides and hydroxides [5,18]. Transition metal sulfides, such as cobalt sulfides and nickel sulfides, have been investigated as a new electrode material for pseudocapacitors with a good performance [11,19]. Particularly, NiCo_2_S_4_ exhibited an electrical conductivity about 100 times that of NiCo_2_O_4_ and approximately 10^4^ times higher than conventional mono-metal counterparts [20–22].

However, the conductivity and the structure stability of the NiCo_2_S_4_ nanomaterials reported are insufficient to meet the requirements for the high-performance supercapacitors. Many strategies have been developed to solve the above challenges, such as constructing special core shell structure [13,20,23], fabricating composite material [11,24], direct growth on conductive substrate [25,26] and so on. Among them, coupling the transition metal sulfides with carbon nanomaterials is an effective means to overcome the drawbacks. For example, carbon nanotubes (CNTs) and graphene have excellent physical properties such as electrical conductivity, large surface area and pore structure [27], which are widely used to integrate with other materials. Wen and his partners have prepared the hybrids of nickel cobalt sulfide/multi-wall carbon nanotubes (NiCo_2_S_4_/MWCNTs) by one-pot solvothermal reaction, which is able to deliver an ultrahigh specific capacitance of 2080 F g^−1^ at the current density of 1 A g^−1^ and exhibit an excellent cycling stability of 85.7% capacitance retention after 5000 cycles at 4 A g^−1^ in an asymmetric supercapacitor [24]; Yang and her cooperators have fabricated edge site-enriched nickel-cobalt sulfide (Ni-Co-S) nanoparticles anchored on graphene frameworks exhibiting a superior rate capability of 96% with the current density increased [28]. Though lots of achievements have been obtained, more effort is still needed to realize our desired high performance for energy storage material. So far, there is little literature to compare the structures and properties of the NiCo_2_S_4_ samples using the different sulfur sources under the same experimental conditions.

In this work, we successfully synthesized NiCo_2_S_4_/CNTs nanocomposites through a hydrothermal method with subsequent sulphurizing process. Meanwhile, we investigated the effect of different sulfide sources on the morphology, structure and performance of final samples. Benefiting from the unique porous structure and compositions, the sample, which was prepared with thioacetamide (TAA) as a sulfide source, showed high specific capacitance and excellent cycling stability. Furthermore, an asymmetric supercapacitor (ASC) was assembled with the NiCo_2_S_4_/CNTs electrode as a positive electrode and graphene as a negative electrode. The device delivered a high energy density of 29.44 W h kg^−1^ at a power density of 812 W kg^−1^. The above results imply that NiCo_2_S_4_/CNTs nanocomposites are a promising electrode material in supercapacitors for practical application.

## 2. Material and methods

### 2.1. Preparation of nickel cobalt oxide nanostructures

All the chemicals were directly used after purchase without further purification. In a typical process, 0.952 g of CoCl_2_·6H_2_O, 0.476 g of NiCl_2_·6H_2_O, 0.37 g of NH_4_F and 44 mg of CNTs were dissolved in 50 ml of deionized (DI) water and 20 ml of alcohol, resulting in homogeneous black solution under vigorous magnetic stirring and ultra-sonication for 20 min. Then, 1.5 g of hexamethylenetetramine (HMT) was added into the black solution and stirred for about 30 min. The mixed solution was transferred into a 100 ml Teflon-lined stainless steel autoclave, which was kept at 95°C for 24 h. After cooling to room temperature, the products were collected by centrifugation and washed with deionized water (DI) several times to remove the residual reactants. The obtained precipitates were dried in a vacuum oven at 60°C for 12 h.

### 2.2. Preparation of nickel cobalt sulfide nanostructures

93 mg of the precursors prepared in the first step and 0.2 M TAA or 0.2 M Na_2_S·9H_2_O were dispersed directly into 12 ml deionized water. Then, the mixed solution was added into a 25 ml Teflon-lined stainless steel autoclave after stirring for about 30 min. Typically, the autoclave was kept at 120°C for 6 h. After cooling to room temperature, the products were collected by centrifugation and washed with DI water several times and dried at 60°C for 24 h. The resulting samples were labelled S1 and S2. The pure NiCo_2_S_4_ was also prepared by the same method except adding the CNTs. The resulting samples were labelled NiCo_2_S_4_-1 and NiCo_2_S_4_-2.

### 2.3. Materials characterizations

The morphologies and structures of the as-obtained samples were characterized by field emission scanning electron microscopy (FE-SEM, Hitachi S-4800), transmission electron microscopy (TEM, Hitachi JEM-2200FS), powder X-ray diffraction (XRD, Ultima IV, Cu Kα radiation) and X-ray photoelectron spectroscopy (XPS, ESCALAB 250Xi, Al Kα radiation). Nitrogen adsorption and desorption measurements were performed on Quadrasorb SI. The specific surface area was calculated using Brunauer–Emmett–Teller (BET) method.

### 2.4. Electrochemical measurements

All electrochemical measurements were performed on an electrochemical workstation (CHI 660D, Shanghai, China) in three-electrode configurations with 2 M KOH aqueous electrolyte. For the preparation of the working electrodes, the as-synthesized active materials were mixed with acetylene black and polyvinylidene fluoride (PVDF) with a mass ratio of 7 : 2 : 1 in N-methyl-2-pyrrolidinone. Then the mixture was coated into the nickel foam (NF) electrodes. After that, the electrodes were dried in an oven at 80°C for 12 h. The dried electrodes were then pressed using a hydraulic press at a pressure of 10 MPa as the working electrodes. The mass loadings of S1 and S2 are about 3.08 mg cm^−2^ and 3.71 mg cm^−2^, respectively. In the three-electrode electrochemical cell, a Pt wire and a Hg/HgO electrode were used as the counter and reference electrodes, respectively. The capacitive performance of the electrodes was evaluated by cyclic voltammetry (CV), chronopotentiometry (CP) and electrochemical impedance spectroscopy (EIS). EIS measurements were carried out using this apparatus over a frequency range of 10 kHz to 0.01 Hz at 0 V with an AC amplitude of 5 mV.

For the CV measurements, the specific capacitance can be calculated according to the following equation:

$$C=\frac{\int I\text{d}V}{2mV\Delta V\;\;},$$

where *m* is the mass of the active materials (*g*), *V* is the scan rate of the CV curves (V s^−1^), Δ*V* is the potential window (V), *I* is the discharge current (A) and ∫⁡IdV is the area of one CV loop.

For the CP measurements, the specific capacitance can be calculated according to the following equation:

$$C=\frac{I\Delta t}{m\Delta V},$$

where *I* is the discharge current (A), Δ*t* is the discharge time (s), *m* is the mass of the electrode materials (g) and Δ*V* is the potential range (V).

## 3. Results and discussion

### 3.1. Sample characterization

**Figure 1.**
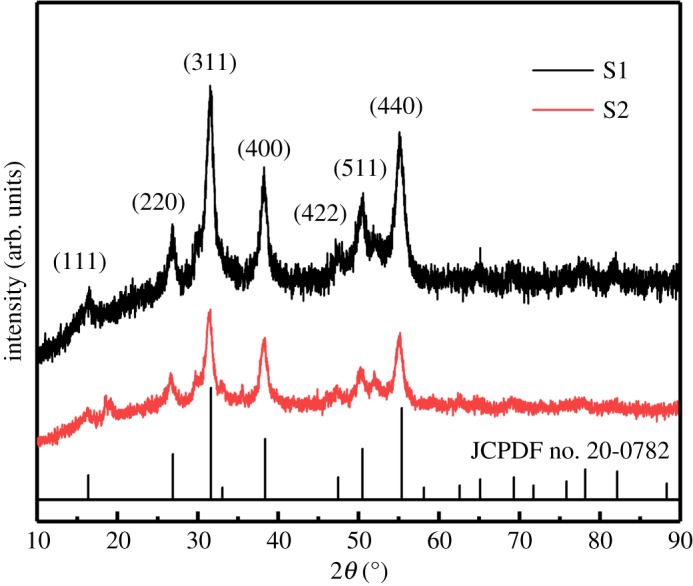
XRD patterns of the S1 and the S2 samples.

The crystal structure and phase of the samples were characterized by XRD. Figure 1 illustrates the XRD patterns of the S1 and the S2 samples. Obviously, the diffraction peaks about at 16.3°, 26.8°, 31.6°, 38.3°, 47.4°, 50.5° and 55.3° of the two samples can be indexed to the (111), (220), (311), (400), (422), (511) and (440) planes of the cubic phase of NiCo_2_S_4_ (JCPDS no. 20-0782), respectively. The XRD pattern of the S1 sample without any impurity phases indicates that the precursor was completely vulcanized into NiCo_2_S_4_. However, some additional peaks of the S2 sample at 18.4° correspond to NiS (JCPDS no. 02-1443), and the peaks at 29.8° and 51.9° for the two samples can be attributed to Co_9_S_8_ (JCPDS no. 02-1459), on account of the incomplete sulphurization of the S2 sample using Na_2_S as sulfur source [29]. It is clear that the peaks of S1 and the S2 were similar which only had slight shifts and changes. Furthermore, the diffraction peaks of the CNTs could not be clearly identified due to their low content and small atomic number [11].

**Figure 2.**
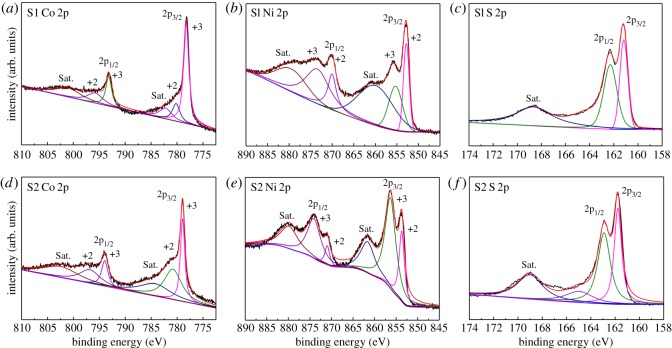
XPS spectra for the (*a*) Co 2p, (*b*) Ni 2p, and (*c*) S 2p for the S1 samples. XPS spectra for the (*d*) Co 2p, (*e*) Ni 2p, and (*f*) S 2p for the S2 samples.

The elemental compositions and chemical bonding states of the NiCo_2_S_4_ were further investigated by X-ray photoelectron spectroscopy (XPS). As shown in the figure 2, the elements of Ni, Co and S were clearly exhibited. For both the two samples, Co 2p and Ni 2p spectra can both be fitted with two spin–orbit peaks and two shake-up satellites (marked as ‘Sat.’) using the Gaussian fitting method. For Co 2p of the S1 sample (figure 2*a*), the binding energies at around 778.2 and 793.2 eV indicate the existence of Co^3+^, while the peaks at around 780.2 and 796.01 eV are assigned to Co^2+^. Likewise, two kinds of Ni species can be observed (figure 2*b*); the fitting peaks at 852.8 and 870.2 eV imply the characteristic of Ni^2+^, where the binding energies at 855.6 and 873.9 eV are indexed to Ni^3+^. These results agree with the literature of the Co 2p and Ni 2p spectra in NiCo_2_S_4_ [11,29]. Moreover, the peak (figure 2*c*) at 161.2 eV is indexed to S 2p_3/2_ associated with the characteristic of metal–sulfur bonds, while the peak at 162.4 eV corresponding to S 2p_1/2_ may be attributed to S^2−^ in low coordination at the surface, which is generally related to sulfur vacancies [25,29,30]. The similar results of the S2 sample can also be observed [31,32]. Moreover, the XPS results coincide well with the XRD results.

**Figure 3.**
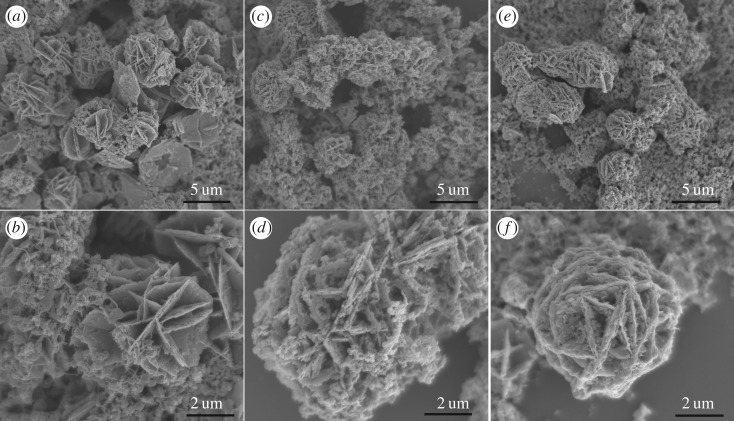
SEM images of the precursors (*a*,*b*), S1 (*c*,*d*) and S2 (*e*,*f*) at low (*a*,*c*,*e*) and high (*b*,*d*,*f*) magnifications.

The morphologies and nanostructures of the samples were examined via scanning electron microscopy (SEM), as shown in figure 3. The morphologies of the predecessors are many nanosheets and the flower-like balls composed of nanosheets (figure 3*a*). No matter whether the sulfur sources are TAA (figure 3*c*,*d*) or Na_2_S · 9H_2_O (figure 3*e*,*f*), the morphology of the NiCo_2_S_4_ structures was well inherited from the precursor after the subsequent sulphuration. Obviously, the difference between the two sulfide products was the surface of the nanosheets. The surface of the S1 samples (electronic supplementary material, figure S6a) was loose and porous compared with S2 (electronic supplementary material, figure S6b). This result was also proved by the pure NiCo_2_S_4_-1 and NiCo_2_S_4_-2 (electronic supplementary material, figure S1). The porosity favours to expose richer redox sites and the electrolyte transport, which can highly facilitate rapid redox reactions and increase the contact area between the electrode and electrolytes for the effective utilization of the active materials [33–35]. Comparing figure 3 with electronic supplementary material, figure S1, it is seen that the morphologies of samples are composed of thick nanoplates without CNTs. It is obvious that adding CNTs can shrink the thickness of nanoplates to form thin nanosheets.

**Figure 4.**
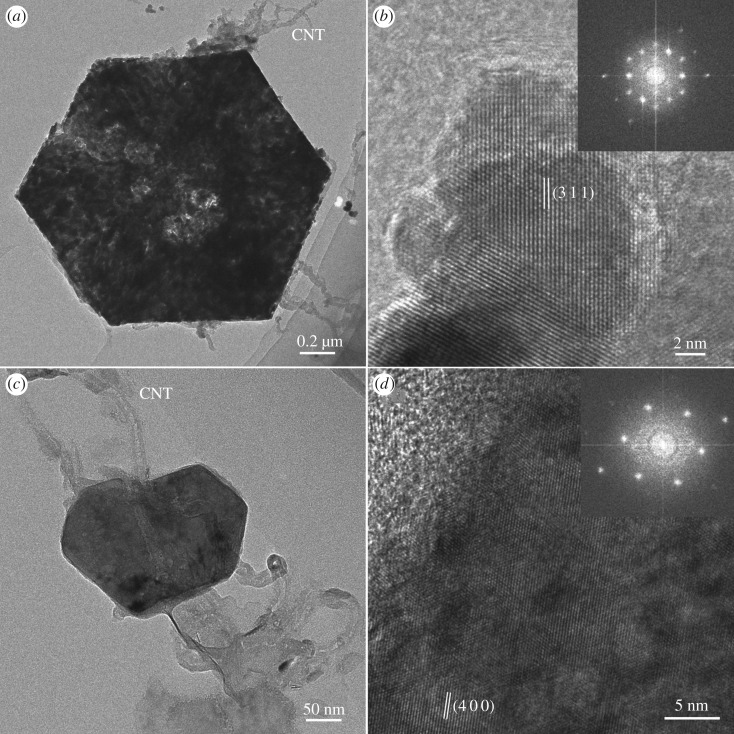
(*a*) and (*b*) TEM and HRTEM images of S1; the inset of (*b*) is the corresponding FFT. (*c*) and (*d*) TEM and HRTEM images of S2; the inset of (*d*) is the corresponding FFT image.

The nanostructures of NiCo_2_S_4_ flower-like balls were further observed by FE-TEM. The morphology of the nanosheets (figure 4*a,c*) was also well inherited from the precursor. Nevertheless, the sample prepared by using TAA as a sulfide source possesses more porous structures, which could provide more active sites and lead to superior electrochemical performance, consistent with the results of the SEM. From 4*a* and 4*c*, it is clear that CNTs can coat well on the surface of nanosheets, indicating that CNTs have played a part in the formation of flower-like balls, acted as a backbone matrix to uphold the structural integrity of the network and played a role as electron channels [36]. In figure 4*b*, the interplanar spacing between the lattice fringe is measured to be 0.283 nm corresponding to the (311) plane of the NiCo_2_S_4_ and the corresponding fast Fourier transform (FFT) pattern (inset in figure 4*b*) confirms its good crystallinity. In figure 4*d*, the measured interplanar spacing of 0.234 nm can be indexed to the (400) plane of the NiCo_2_S_4_, and the corresponding FFT pattern (inset in figure 4*d*) also confirms its good crystallinity. These results are in good agreement with the XRD results (figure 1).

The specific surface area (SSA) and the distribution of the pore size of the electrode materials can be the judgements of the electrochemical performances. S1 and S2 samples were explored by recording the adsorption–desorption isotherms of nitrogen at 77 K. As shown in electronic supplementary material, figure S2, the SSA of S1 and S2 by using the BET method are calculated to be about 32.82 and 19.7 m^2^ g^−1^, respectively. The large SSA is beneficial for substantial contact between the electrolyte and the active material, which can improve the electrochemical performances.

### 3.2. Electrochemical properties

**Figure 5.**
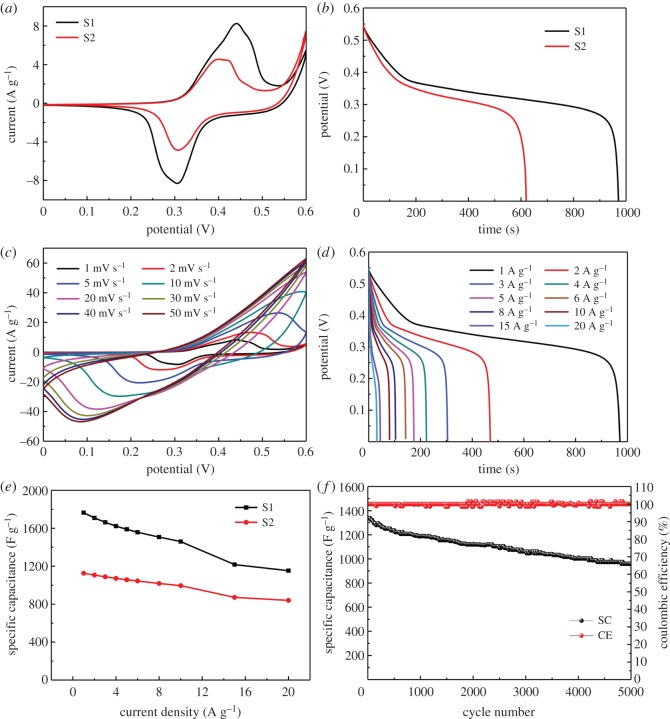
(*a,b*) CV curves and Galvanostatic discharge curves of the S1 and S2 electrodes measured at 1 mV s^−1^ and 1 A g^−1^. (*c*) CV curves of the S1 electrodes at different scan rates and (*d*) Galvanostatic discharge curves at various current densities. (*e*) The specific capacitance versus current density for the S1 and S2 electrodes. (*f*) Cycling performance and coulombic efficiency of the prepared S1 electrodes over 5000 charge–discharge cycles at a constant current density of 10 A g^−1^.

In order to characterize the capacitor behaviour of electrode materials, the electrochemical performances of the as-synthesized samples as a positive electrode were investigated in a three-electrode system. The S1 and S2 electrodes were tested under the same conditions for comparison. Figure 5*a* compares the CV curves of the S1 with S2 electrodes obtained at a scan rate of 1 mV s^−1^. Obviously, the integrated area covered by the CV curves of the S1 sample was much larger than the S2 sample, suggesting that the S1 sample has a superior electrochemical performance. The higher pseudocapacitive performance can be attributed to the loose and porous surface, which is conducive to the contact between the electrode and electrolytes.

Figure 5*b* compares the Galvanostatic discharge curves of the S1 and S2 electrodes at the same current density of 1 A g^−1^. The discharging time for the S1 (971 s) is much higher than the S2 (619.6 s), which also demonstrates that the S1 sample has higher electrochemical performance. To confirm the importance of CNTs, the CV curves and Galvanostatic discharge curves of the pure NiCo_2_S_4_-1 (514.6 s) and the pure NiCo_2_S_4_-2 (402.7 s) electrodes were also measured at 1 mV s^−1^ and 1 A g^−1^ as a contrast (electronic supplementary material, figure S3). The results reveal that the samples with CNTs have better electrochemical performance than samples without CNTs. In the meantime, it is also concluded that using TAA as the sulfur source can have a better electrochemical performance than Na_2_S·9H_2_O as sulfur source.

The energy storage of the NiCo_2_S_4_ electrode is ascribed to the Faradaic redox reactions of NiCo_2_S_4_ in 2 M KOH electrolyte. Figure 5*c* shows cyclic voltammetry (CV) curves of the S1 electrode at various scan rates ranging from 1 to 50 mV s^−1^. As the scan rate increases, the anodic and cathodic peaks move to the positive and negative potential. The obvious redox reaction peaks in the CV curves indicate that distinct pseudocapacitive characteristics of the electrode materials are mainly governed by Faradaic redox reactions, which can be originated from reversible redox processes of Ni^2+^/Ni^3+^ and Co^2+^/Co^3+^/Co^4+^ [29,37]. The plausible electrochemical reactions are described as follows:

$$\text{NiC}{\text{o}}_{2}{\text{S}}_{4}\;\;+\;O{\text{H}}^{-}+{\text{H}}_{2}\text{O}↔\text{NiSOH + 2CoSOH + 2}{\text{e}}^{-}$$

$$\text{CoSOH + O}{\text{H}}^{-}↔\text{CoSO + }{\text{H}}_{2}\text{O + }{\text{e}}^{-}$$

Figure 5*d* further investigates the discharge times of the S1 samples at current densities from 1 to 20 A g^−1^. According to the curves, the specific capacitances of the S1 electrodes are 1765, 1709, 1663, 1623, 1589, 1558, 1507, 1460, 1217 and 1153 F g^−1^ at the current densities of 1, 2, 3, 4, 5, 6, 8, 10, 15 and 20 A g^−1^, respectively. Moreover, the capacitance at 10 A g^−1^ corresponds to 82.7% retention relative to 1 A g^−1^. Figure 5*e* shows the corresponding specific capacitances versus discharge current density of the S1 and S2 electrode. Apparently, the S1 demonstrates the higher specific capacitance than the S2. Meanwhile, the results reveal that the specific capacitance decreases as the current density increases, which occurs because the redox reaction between Ni/Co cations and OH anions is a diffusion-controlled process through the electrode grain boundaries. However, at a current density of 20 A g^−1^, the rate capability of the S2 samples was 74.6% retention, which was higher than that of S1 samples (65.3%).

To check the structural stability and corresponding coulombic efficiency of the prepared S1 electrodes, a cycling test was performed over 5000 charge–discharge cycles at a constant current density of 10 A g^−1^ (figure 5*f*). The specific capacitance remained approximately 71.7% of the initial value after 5000 charging–discharging cycles, thus showing excellent structural stability.

To further investigate the electrical conductivity of the samples, electrochemical impedance spectroscopy (EIS) was measured at an open circuit potential in the frequency range from 100 kHz to 0.01 Hz with an amplitude of 5 mV. Electronic supplementary material, figure S4 presents the Nyquist plots of the different electrodes with an enlarged view (inset). All the curves consist of the intersection on the real axis related to the internal resistances of the electrode (Rs), the semicircle corresponding to the charge transfer resistance (Rct) at a high frequency, and the linear slope meaning the diffusive resistance of the capacitor behaviour at a low frequency [38]. Obviously, the Nyquist plots of the two samples present semicircles with a small diameter in a high frequency range, indicating low charge-transfer resistance. From the point intersecting with the real axis, the internal resistances of samples were approximately 0.59 and 0.71 Ω. As the previous revealed, it is notable that the trend of conductivity is well consistent with the capacitance, which can imply that there is a strong correlation between the electrical conductivity and the specific capacitance. The results demonstrate that the S1 samples can provide a better supercapacitive performance.

**Figure 6.**
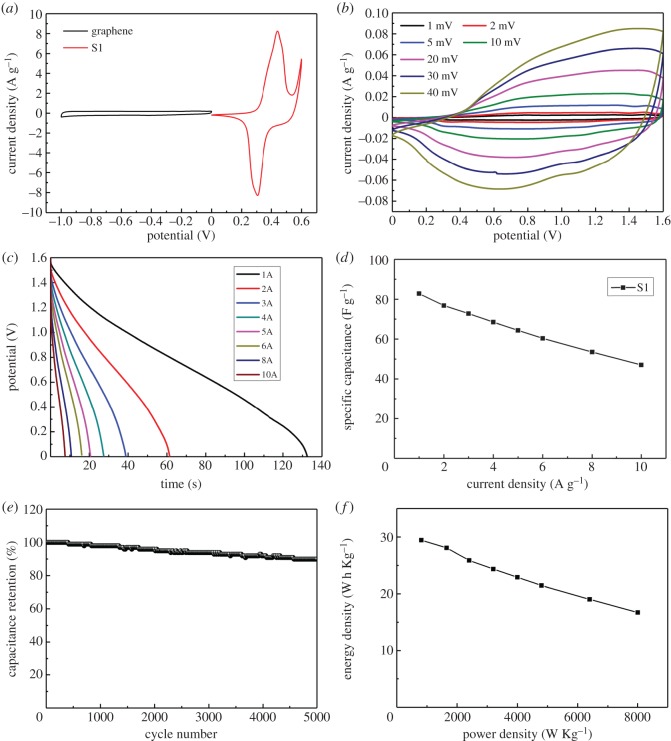
(*a*) CV curves of the graphene electrode and the S1 electrode at 1 mV s^−1^. (*b*) CV curves of the S1//graphene ASC measured at different scan rate in the potential window of 0–1.6 V. (*c*) Galvanostatic discharge curves of the S1//graphene ASC at different current densities. (*d*) The specific capacitance of the S1//graphene ASC at different current densities. (*e*) Cycling stability of the S1//graphene ASC at a constant current density of 5 A g^−1^. (*f*) Ragone plot of the S1//graphene ASC from the Galvanostatic charge–discharge curves.

To further explore the potential practical application of the S1 electrodes in the energy field, an asymmetric supercapacitors (ASC) device was successfully fabricated with the S1 electrode as the positive electrode and graphene as the negative electrode in 2 M KOH electrolyte. With the discovery of graphene, it is well known that graphene (electronic supplementary material, figure S5) is an ideal supercapacitor electrode material because of its high specific surface area, good conductivity and excellent electrochemical stability. The mass loading of graphene is determined by balancing the charge between both electrodes. Figure 6*a* presents the CV curves of the graphene electrode and the S1 electrode at 1 mV s^−1^. The CV curves of the graphene exhibit a nearly rectangular shape without redox peaks, which imply the excellent EDLC performance with a potential window from −1.0 to 0 V. As for S1 electrode in the voltage window from 0 to 0.6 V, two pairs of redox peaks are observed, owing to the classic pseudocapacitance. According to the previous researches, we calculated the optimal mass ratio m (S1)/m (graphene) ≈0.2 based on their specific capacitances and potential windows, which can obtain the maximum capacitance of the device and meet the requirement of the charge balance between both electrodes.

Figure 6*b* shows CV curves of S1//graphene in a voltage window of 0–1.6 V at different scan rates in a 2 M KOH electrolyte. The ASC curves show a quasi-rectangular shape with redox peaks, indicating that the whole capacitance can be attributed to the combination of ELDC and Faradaic pseudocapacitance. The capacitances calculated based on the CV curves show that the ASC provides a high capacitance of 100 F g^−1^ at 1 mV s^−1^ scan rate and still exhibits 80 F g^−1^ at a high scan rate of 50 mV s^−1^. The capacitance drop is generally caused by the insufficient time for the electrolyte to access the electrode surface.

Figure 6*c* presents the Galvanostatic charge–discharge measurements at various current densities from 1 to 10 A g^−1^ in the potential window of 1.6 V. The ASC exhibits a high specific capacitance of 82.8 F g^−1^ at the current density of 1 A g^−1^. With the current density increasing to 10 A g^−1^, the specific capacitance still remains 46.9 F g^−1^, which is 56.6% of the capacitance retained. The cycle stability of the ASC device was valued by the GCD measurements at a high current density of 5 A g^−1^ for 5000 cycles (figure 6*e*). Remarkably, only 10.2% capacity decay is observed after 5000 cycles.

Another essential factor to demonstrate the electrochemical performance of devices is energy density. Figure 6*f* presents the Ragone plot of the ASC from the Galvanostatic charge–discharge curves. The ASC achieved a high energy density of 29.44 W h kg^−1^ at a power density of 812 W kg^−1^ and can retain 16.68 W h kg^−1^ at a higher power density of 8006 W kg^−1^. This high energy density may be attributed to its high specific capacitance and the good match of NiCo_2_S_4_/CNTS with graphene. The above results indicate that the NiCo_2_S_4_/CNTS is a promising candidate material in supercapacitors field.

## 4. Conclusion

In summary, we have successfully synthesized NiCo_2_S_4_/CNTS nanocomposites via a simple hydrothermal method with subsequent sulphurizing process. By comparing two different sulfur sources, the S1 sample using TAA as sulfur source possesses more pure phase and exhibits a higher capacitance of 1765 F g^−1^ than S2 electrodes with 1250 F g^−1^ at a current density of 1 A g^−1^, which might be attributed to its loose and porous structure. Moreover, an ASC device based on NiCo_2_S_4_/CNTS as the positive electrode and graphene as the negative electrode has been assembled. It achieved a high specific capacitance of 82.8 F g^−1^ at a current density of 1 A g^−1^ with a potential window of 1.6 V. Furthermore, it can deliver a remarkable energy density of 29.44 W h kg^−1^ at a power density of 812 W kg^−1^. Additionally, the ASC device exhibits good cycling stability (89.8% retaining after 5000 cycles). This study indicates their potential as a promising candidate material for high-performance supercapacitors.

## Supplementary Material

Facile Synthesis of NiCo2S4/CNTs Nanocomposites for High-Performance Supercapacitors

## Data accessibility

We have deposited our data in the Dryad Digital Repository (http://dx.doi.org/10.5061/dryad.nf1kf60) [39]. The datasets supporting this article have been uploaded as part of the electronic supplementary material.
