# Supplementary material for: Facile synthesis of NiCo2S4/CNTs nanocomposites for high-performance supercapacitors
Source: R Soc Open Sci. 2018 Sep 12;5(9):180953. doi: 10.1098/rsos.180953 (PMC6170541; doi:10.1098/rsos.180953)
Supplement: Facile Synthesis of NiCo2S4/CNTs Nanocomposites for High-Performance Supercapacitors [file rsos180953supp1.docx]

**
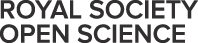
**

Supplementary Information

Facile Synthesis of NiCo_2_S_4_/CNTs Nanocomposites for High-Performance Supercapacitors

Yunxia Huang, Ming Cheng, Zhongcheng Xiang and Yimin Cui *

Department of Physics, Beihang University, Beijing 100191, P. R. China

The electronic supplementary information we prepared is the results of the SEM morphologies of the pure NiCo_2_S_4_, N_2_ adsorption and desorption isotherms and the pore-size-distribution curves, the CV curves and Galvanostatic discharge curves of the pure NiCo_2_S_4_-1 and the pure NiCo_2_S_4_-2 electrodes measured at 1 mV/s and 1 A/g, Nyquist plots, SEM image of the graphene, SEM image of the S1 and S2 sample at high-magnifications as shown in Figure S1, S2, S3, S4, S5 and S6.


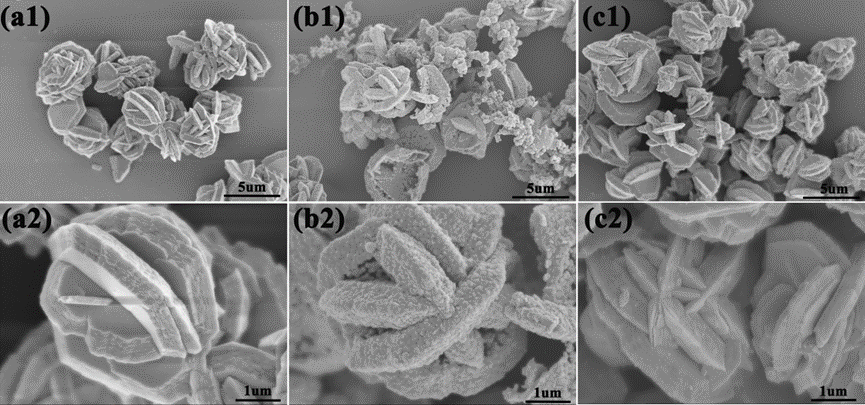


**Figure S1** SEM images of the precursors (a1 and a2), the pure NiCo_2_S_4_-1 (b1 and b2) and NiCo_2_S_4_-2 (c1and c2) at low- and high-magnifications, respectively.


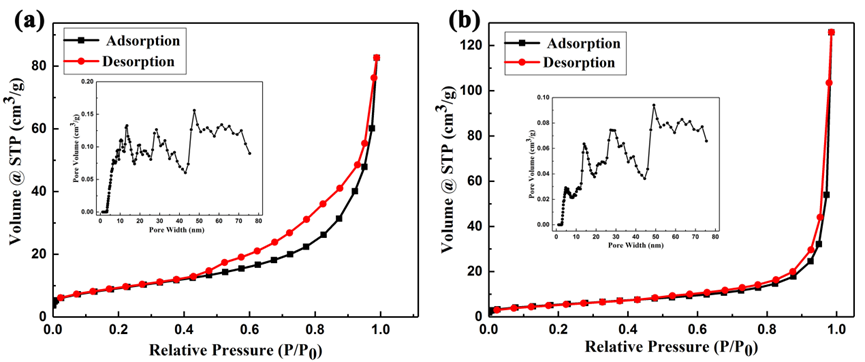


**Figure S2** (a) and (b) Typical N_2_ adsorption and desorption isotherms and the pore-size-distribution curves (inset) of the S1 and S2, respectively.


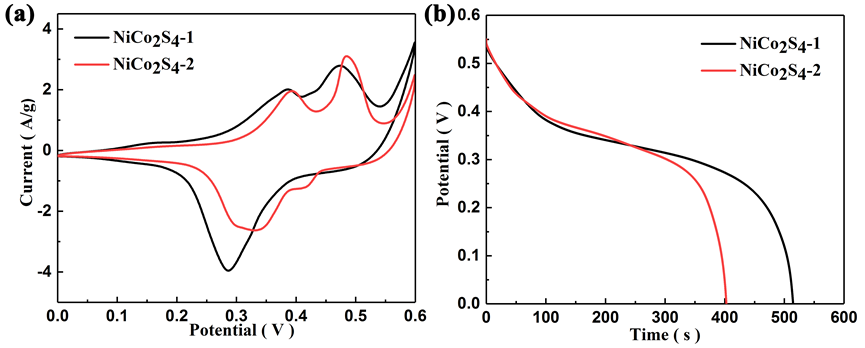


**Figure S3** CV curves and Galvanostatic discharge curves of the pure NiCo_2_S_4_-1 and the pure NiCo_2_S_4_-2 electrodes measured at 1 mV/s and 1 A/g.





**Figure S4** EIS Nyquist plots of the S1 and S2 electrode


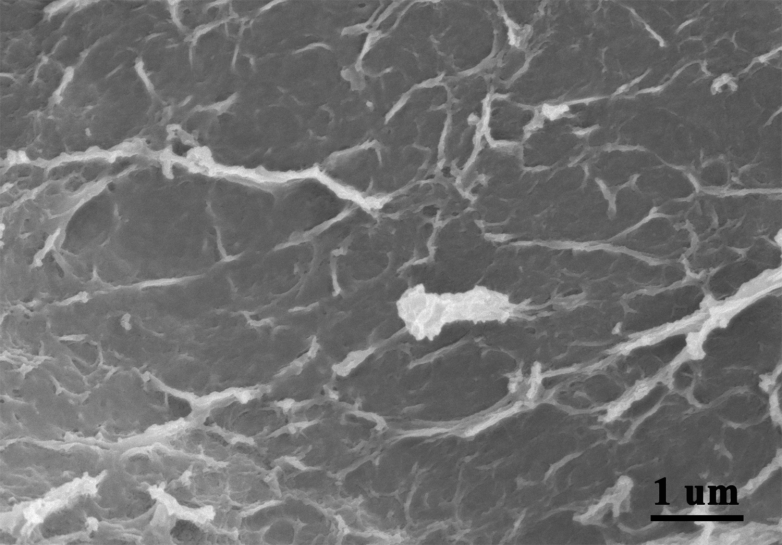


**Figure S5** SEM image of the graphene.


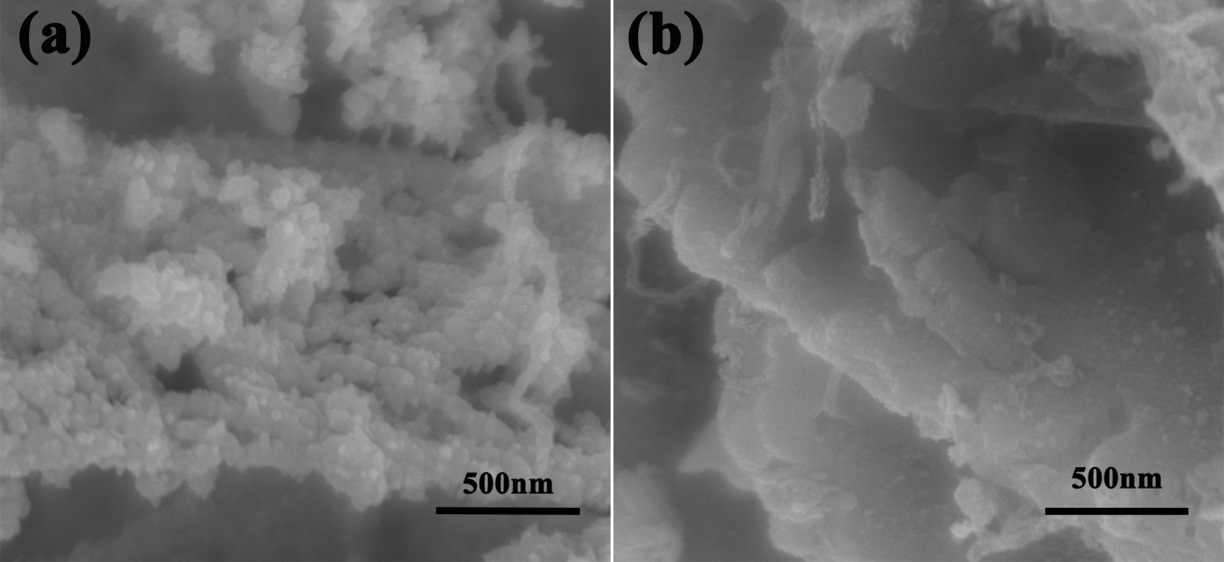


**Figure S6** SEM image of the S1 (a) and S2 (b).
